# Preoperative serum levels of HE4 and CA125 predict primary optimal cytoreduction in advanced epithelial ovarian cancer: a preliminary model study

**DOI:** 10.1186/s13048-020-0614-1

**Published:** 2020-02-12

**Authors:** Li-yuan Feng, Sheng-bin Liao, Li Li

**Affiliations:** Department of Gynecologic oncology, Guangxi Medical University Cancer Hospital, 71 Hedi Road, Nanning, Guangxi 530021 People’s Republic of China

**Keywords:** Primary optimal cytoreduction, Noninvasive prediction model, Preoperative HE4 level, Preoperative CA125 level

## Abstract

**Objective:**

The aim of this study is to establish a noninvasive preoperative model for predicting primary optimal cytoreduction in advanced epithelial ovarian cancer by HE4 and CA125 combined with clinicopathological parameters.

**Methods:**

Clinical data including preoperative serum HE4 and CA125 level of 83 patients with advanced epithelial ovarian cancer were collected. The sensitivity, specificity, positive predictive value, negative predictive value and overall accuracy of each clinical parameter were calculated. The Predictive Index score model and the logistic model were constructed to predict the primary optimal cytoreduction.

**Results:**

Optimal surgical cytoreduction was achieved in 62.65% (52/83) patients. Cutoff values of preoperative serum HE4 and CA125 were 777.10 pmol/L and 313.60 U/ml. (1) Patients with PIV ≥ 6 may not be able to achieve optimal surgical cytoreduction. The diagnostic accuracy, NPV, PPV and specificity for diagnosing suboptimal cytoreduction were 71, 100, 68, and 100%, respectively. (2) The logistic model was: logit *p* = 0.12 age − 2.38 preoperative serum CA125 level − 1.86 preoperative serum HE4 level-2.74 histological type-3.37. AUC of the logistic model in the validation group was 0.71(95%CI 0.54–0.88, *P* = 0.025). Sensitivity and specificity were 1.00 and 0.44, respectively.

**Conclusion:**

Age, preoperative serum CA125 level and preoperative serum HE4 level are important non-invasive predictors of primary optimal surgical cytoreduction in advanced epithelial ovarian cancer. Our PIV and logistic model can be used for assessment before expensive and complex predictive methods including laparoscopy and diagnostic imaging. Further future clinical validation is needed.

## Introduction

Ovarian cancer is the most lethal gynecological malignancy. More than 75% of ovarian cancer patients are in stage III-IV at the time of initial diagnosis. The 5-year survival rate is less than 30% [1]. Residual lesions after primary surgery remain one of the most important prognostic factors in patients with advanced ovarian cancer [2–5].

Models predicting the primary optimal surgical debulking are important in guiding the initial treatment of patients with advanced ovarian cancer. Unsatisfactory primary debulking surgery (PDS) does not improve the prognosis, and may increase perioperative morbidity [6, 7]. Advanced ovarian cancer patients who are older, with increased comorbidities, and with a higher disease burden who cannot achieve primary optimal surgical debulking can obtain the similar overall survival rates and lower postoperative adverse events compared to patients with optimal cytoreduction when they choose neoadjuvant chemotherapy [7–10]. Significant variability in the rate of primary optimal surgical cytoreduction for advanced ovarian cancer, ranging from 25 to 90%, exists between institutions [11]. Models predicting the primary optimal surgical debulking are popular, but maddening topic. There are many variables which include tumor volume and stage, patient performance status and ability to tolerate long and complex operations, and surgeons’ skills make predictions difficult. Several attempts have been made to predict primary surgical outcome using imaging methods, CA125, and laparoscopic scoring. These studies have been limited by a lack of sensitivity or specificity, complex models, and broad criteria for inclusion.

The ideal preoperative prediction model should accurately identify the optimal and suboptimal surgical outcome, have wide applicability, be accurate and be non-invasive. CA125 and HE4 are considered to be the most promising serum markers of ovarian cancer [12–17]. The purpose of our study was to explore whether the combination of preoperative serum CA125 level and preoperative serum HE4 level could establish a reliable noninvasive preoperative predictive score to assess resectability in initial treatment of advanced ovarian cancer.

## Materials and methods

### Study population

**Fig. 1:**
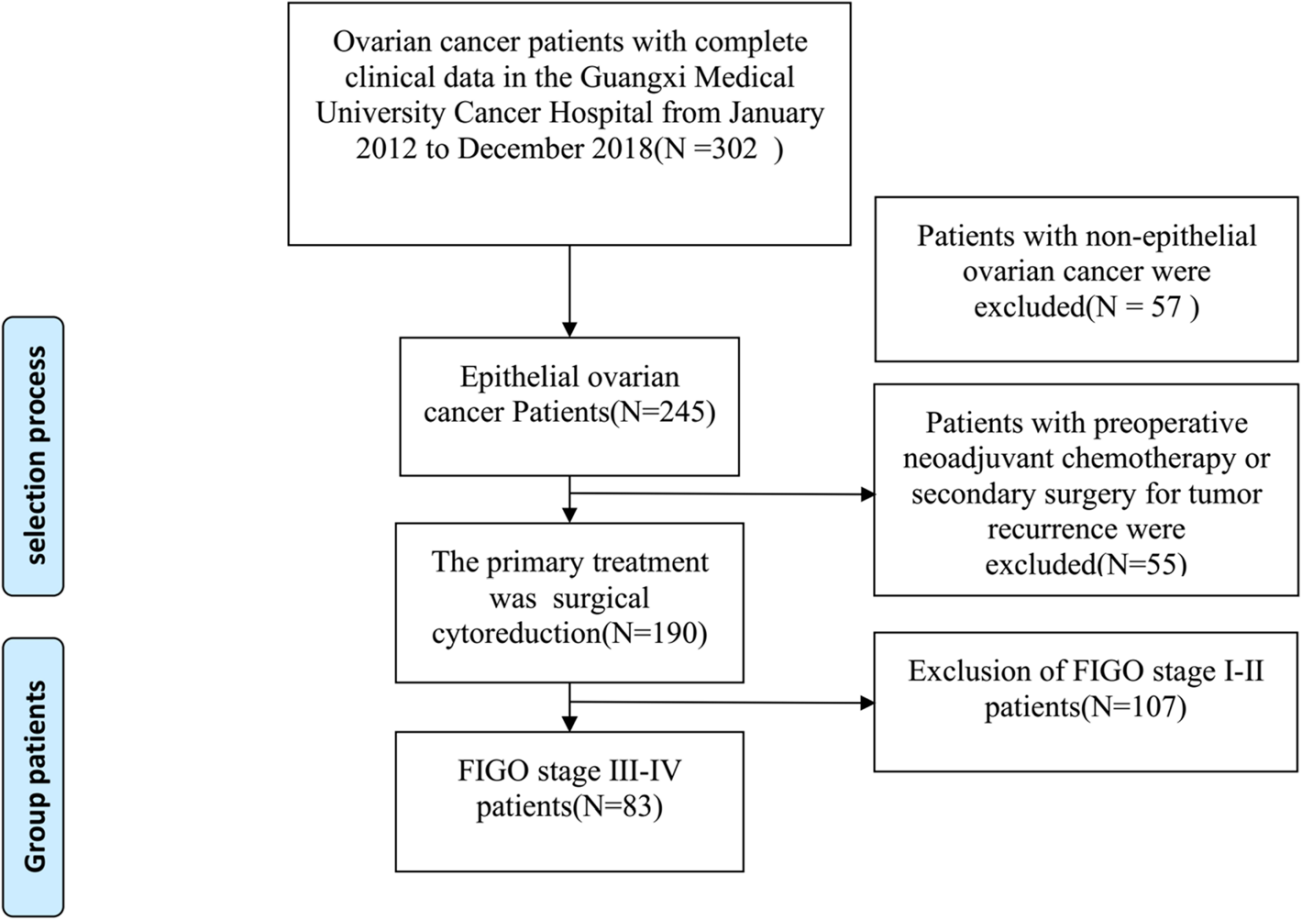
Flow chart of patient selection

A retrospective analysis of ovarian cancer patients in the Guangxi Medical University Cancer Hospital from January 2012 to December 2018 was performed. The inclusion and exclusion criteria are as follows. Inclusion criteria: 1) patients with complete clinical data (including: age, preoperative serum CA125 level, preoperative serum HE4 level, histological type, FIGO stage, grade, Eastern Cooperative Oncology Group (ECOG) performance status, American Society of Anesthesiologists (ASA) and surgical outcome); 2) patients with ovarian epithelial carcinoma confirmed by pathology; 3) initial treatment of primary surgical debulking; 4) FIGO stage III or IV. Exclusion criteria: 1) patients without complete clinical data; 2) non-ovarian epithelial cancer patients confirmed by pathology; 3) patients undergoing preoperative neoadjuvant chemotherapy or a second operation for tumor recurrence; 4) FIGO stage I or II. The patient selection process is shown in Fig. 1. Eighty-three patients met the inclusion criteria. The primary optimal surgical cytoreduction is defined as tumor residue = 0. All patients provided written informed consent and were approved by the institutional review committee of our hospital.

### Statistical analysis

SPSS17.0 was used for analysis. The comparison between the two groups was performed with a t test (metering data) and a chi square test (classification data). The correlation between surgical outcome and clinicopathological parameters was assessed with a Spearman test. The cut-off value of serum HE4 and CA125 was determined with a ROC curve. Sensitivity was defined as the number of suboptimal surgical cytoreduction patients who were correctly identified divided by the total number of suboptimal surgical cytoreduction patients. Specificity was defined as the number of optimal surgical cytoreduction patients who were correctly identified divided by the total number of optimal surgical cytoreduction patients. Positive predictive value corresponded to the number of true positives (suboptimal surgical cytoreduction) divided by the total number of patients predicted to have residual disease, and Negative predictive value corresponded to the number of true negatives (optimal surgical cytoreduction) divided by the total number of patients predicted to have no residual disease. Accuracy was calculated as the sum of the true positives and true negatives divided by the total number of patients in the study. Logistic regression analysis was used to find factors associated with optimal debulking and to establish a logistic model. The Hosmer-Leme test was used to assess the goodness of fit. *P* values were two-sided, and *P* < 0.05 was considered statistically significant. In order to guarantee an adequate significance of the results, the study power was calculated by PASS11.

## Results

### Characteristics of patients

From January 2012 to December 2018, 83 patients met the inclusion criteria. Optimal surgical cytoreduction was achieved in 62.65% (52/83) of patients, the clinical characteristics of the patients are shown in Table 1. The mean age of patients was 53.75 ± 10.51 years. According to FIGO standard staging, there were 74 cases in stage III and 9 cases in stage IV. 68 (81.93%) had disease of serous histology; 55(66.27%)had grade 3. Preoperative serum CA125 level was 1260.84 ± 1542.01 U/ml, and preoperative serum HE4 level was 635.65 ± 749.15 pmol/L. 69 (83.13%) of 83 had an ECOG performance status of 0 and ASA class 2; Preoperative serum HE4 level in patients with suboptimal cytoreduction was significantly higher than that in patients with optimal cytoreduction. The differences in age, FIGO stage, grade, histological type, preoperative serum CA125 level, ECOG performance status, and ASA class between optimal cytoreduction and suboptimal cytoreduction groups were not statistically significant.

**Table 1:** Clinical characteristics of patients were compared according to surgical outcome

Clinical characteristics	All (83)	Optimal (52)	Suboptimal (31)	*P*
Age	53.75 ± 10.51	52.13 ± 9.81	56.45 ± 11.24	0.070
FIGO stage				
III	74 (89.16%)	46 (88.46%)	28 (90.32%)	0.791
IV	9 (10.84%)	6 (11.54%)	3 (9.68%)	
Tumor grade				
1–2	28 (33.73%)	18 (34.62%)	10 (32.26%)	0.826
3	55 (66.27%)	34 (65.38%)	21 (67.74%)	
Histology				
Serous	68 (81.93%)	45 (86.54%)	23 (74.19%)	0.157
Others	15 (18.07%)	7 (13.46%)	8 (25.81%)	
Preoperative serum CA125 level(U/ml)	1260.84 ± 1542.01	1285.57 ± 1662.24	1219.36 ± 1341.36	0.851
Preoperative serum HE4 level (pmol/L)	635.65 ± 749.15	419.96 ± 355.56	997.44 ± 1050.33	0.006^*^
ECOG performance status				
0	69 (83.13%)	45 (86.54%)	24 (77.42%)	0.131
1	9 (10.84%)	6 (11.54%)	3 (9.68%)	
2	5 (6.03%)	1 (1.92%)	4 (12.90%)	
ASA				
1	9 (10.84%)	5 (9.61%)	4 (12.90%)	0.886
2	69 (83.13%)	44 (84.62%)	25 (80.65%)	
≥ 3	5 (6.03%)	3 (5.77%)	2 (6.45%)	

Continuous data are represented by means ± standard deviations, while classified data are represented by values and percentages

“*” means *P* < 0.05

### Diagnostic efficacy of clinicopathological parameters predicting suboptimal cytoreduction

Spearman correlation analysis showed that surgical outcome was correlated with age, preoperative serum CA125 level and preoperative serum HE4 level (The correlation coefficients were 0.27, 0.24 and 0.41, respectively). Optimal cytoreduction was more difficult in patients who were older or with higher preoperative serum CA125 and HE4 levels. There was no significant correlation between other clinicopathological parameters and surgical outcome.

ROC analysis revealed cut-off values of serum HE4 and CA125 for predicting suboptimal surgical cytoreduction to be 777.10 pmol/L and 313.60 U/ml. AUC were 0.68(*P* = 0.007) and 0.53(*P* = 0.621), respectively. We concluded that preoperative serum HE4 was better than CA125 in predicting suboptimal surgical cytoreduction. The sensitivity, specificity, PPV and NPV of each clinical parameter independently predicted suboptimal surgical cytoreduction were shown in Table 2.

**Table 2:** Diagnostic efficacy of clinicopathological parameters predicting suboptimal debulking

Clinical characteristics	Cutoff	Sensitivity	Specificity	PPV	NPV	Accuracy	AUC	*P*	Point
Age	68.5	0.16	0.98	0.83	0.66	0.67	0.57	0.281	2.00
FIGO stage	–	0.10	0.89	0.33	0.62	0.59	0.49	0.888	1.00
Histology	–	0.26	0.87	0.53	0.66	0.64	0.56	0.349	2.00
Tumor grade	–	0.68	0.35	0.38	0.64	0.47	0.51	0.858	1.00
Preoperative serum CA125 level(U/ml)	313.60	0.81	0.42	0.45	0.79	0.57	0.53	0.621	1.00
Preoperative serum HE4 level (pmol/L)	777.10	0.48	0.89	0.71	0.74	0.73	0.68	0.007*	2.00
ECOG performance status	0	0.23	0.87	0.50	0.65	0.63	0.55	0.489	2
ASA	3	0.07	0.94	0.40	0.63	0.61	0.50	0.959	1

* means *P* < 0.05

### PIV model for predicting suboptimal surgical cytoreduction

**Fig. 2:**
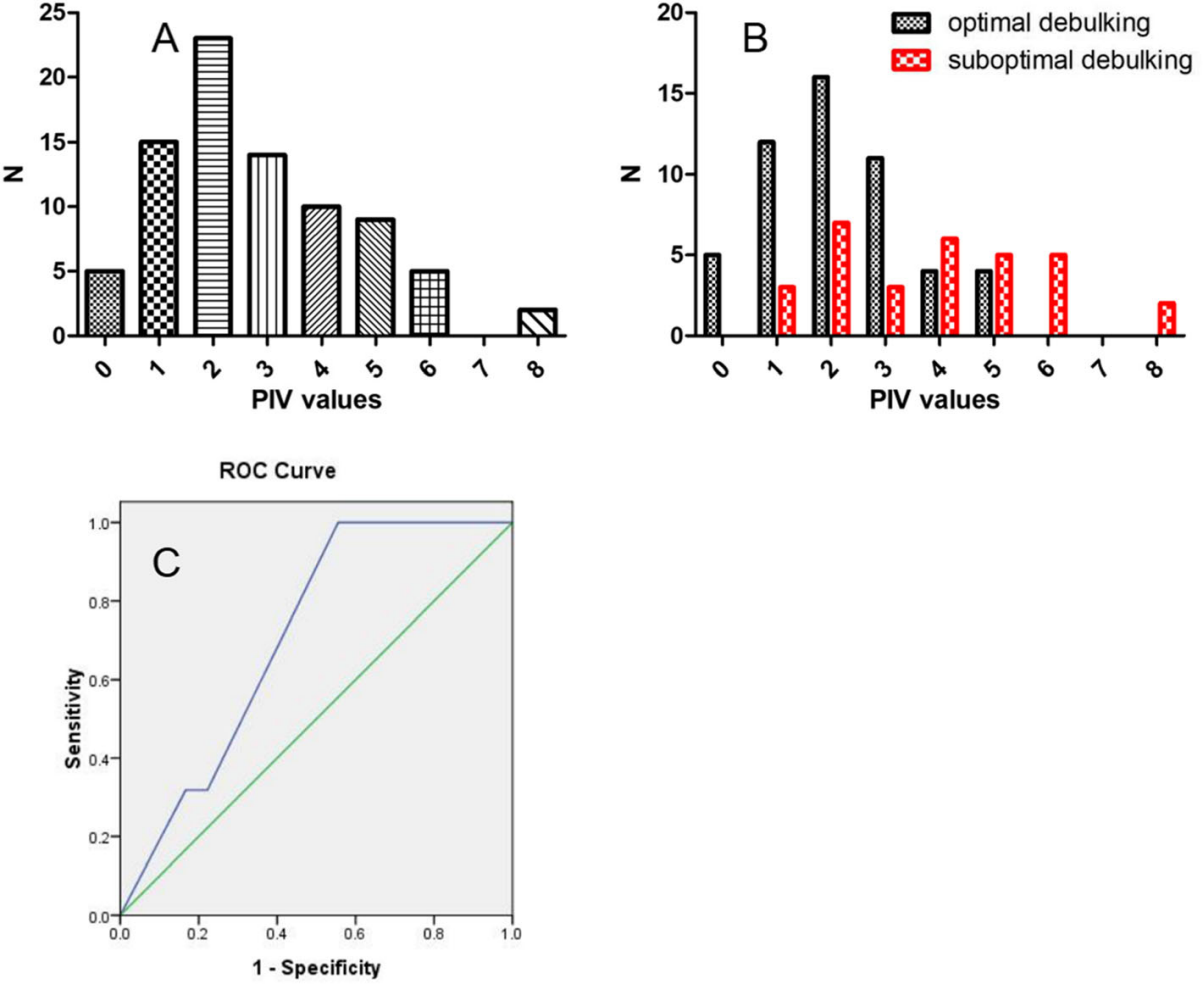
**a**: Number frequency of PIV model score. **b**: Relationship between PIV model score and optimal debulking. **c**: The ROC curve of logistic model in verification set

The parameters that meet the accuracy≥75%, PPV ≥ 50%, and NPV ≥ 50% are included in the predictive index value (PIV) model, and each parameter is assigned 1 point (Table 2). The PIV of each patient was calculated based on the sum of the parameter weights, with a score ranging from 0 to 8. The frequency of individual predictive index score was single peak distribution. Two score was peak (Fig. 2a). The frequency distribution of surgical outcome of each score is shown in Fig. 2b. After PIV > 3, the number of suboptimal surgical cytoreduction patients increased, while the number of optimal surgical cytoreduction patients decreased.

The PIV model shows that with the increase of the score, the diagnostic sensitivity becomes lower and the specificity becomes higher (Table 3). When patient PIV score ≥ 6 points, the accuracy, NPV, PPV and specificity for diagnosing suboptimal cytoreduction were 71%, 100, 68, and 100%. PIV ≥ 6 (7 cases) predicted that patients who could not achieve optimal cytoreduction did not achieve optimal cytoreduction. PIV < 6 predicts that 76 patients can achieve optimal cytoreduction, and 52 patients finally achieve optimal cytoreduction.

**Table 3:** Diagnostic efficacy of each value of PIV model

PIV	Sensitivity(%)	Specificity(%)	NPV(%)	PPV(%)	Accuracy(%)
≥1	100	10	40	100	43
≥2	90	33	47	100	54
≥3	68	63	53	77	65
≥4	58	85	69	77	75
≥5	39	92	75	72	72
≥6	23	100	100	68	71
≥8	6.5	100	100	64	65

### Logistic model for predicting suboptimal surgical cytoreduction

Univariate logistic regression results showed that age, preoperative serum HE4 level and preoperative serum CA125 level were the influencing factors for primary optimal surgical cytoreduction. Age > 69 years (OR = 0.10, 95% CI 0.01–0.92, *P* = 0.042), preoperative serum CA125 level ≥ 313.60 U/ml (OR = 0.33, 95% CI 0.12–0.93, *P* = 0.037), preoperative serum HE4 level ≥ 777.10 pmol/L (OR = 0.14, 95% CI 0.05–0.42, *P* = 0.000) significantly increased the suboptimal cytoreduction. Univariate regression analysis of other clinicopathological factors showed no statistically significant differences.

We constructed a logistic model to predict the optimal surgical cytoreduction. The data was divided into two groups using a random table method (about 1:1): training set (*N* = 43) and verification set (*N* = 40). In the training set, age, FIGO stage, histological type, grade, ECOG performance status, ASA, preoperative serum CA125 level and preoperative serum HE4 level were included to predict the surgical outcome. The results showed that age > 69 years, preoperative serum CA125 level ≥ 313.60 U/ml, preoperative serum HE4 level ≥ 777.10 pmol/L, Serous histological type significantly increased the suboptimal surgical cytoreduction (Age > 69 years (OR = 1.13, 95%CI 1.02–1.26, *P* = 0.024), preoperative serum CA125 level ≥ 313.60 U/ml (OR = 0.09, 95%CI 0.01–0.99, *P* = 0.049), preoperative serum HE4 level ≥ 777.10 pmol/L (OR = 0.16, 95%CI 0.03–0.98, *P* = 0.047), Serous histological type (OR = 0.07, 95%CI 0.01–0.57, *P* = 0.013)). Other clinical parameters were not statistically significant. Logistic regression model was as follows: logit *p* = 0.12 age − 2.38 preoperative serum CA125 level − 1.86 preoperative serum HE4 level-2.74 histological type-3.37. Hosmer-Leme test showed *P* = 0.54, it indicates that the model fits the data better.

In the verification set, the AUC value of logistic model predicting the suboptimal surgical cytoreduction was 0.71(95%CI 0.54–0.88), *P* = 0.025. The sensitivity and specificity were 1.00 and 0.44. The ROC curve was shown in Fig. 2c. With a sample size of 83, the study would have 96% power to meet the AUC = 0.73 if the optimal surgical cytoreduction rate was 62.65%.

### Literature reviews of preoperative serum HE4 predicting the primary optimal surgical cytoreduction

Almost all studies assessing the predictive value of serum HE4 level in predicting surgical outcome of ovarian cancer have confirmed the effectiveness of HE4. We reviewed the study of HE4 in predicting the primary optimal surgical cytoreduction, and compared the diagnostic efficacy of each study. Results are shown in Table 4.

**Table 4:** Literature reviews of HE4 predictive diagnostic efficacy for primary optimal surgical debulking

Year	Author	N	Country	Optimal surgical debulking rate	HE4 cutoff value (pmol/L)	Sensitivity	Specificity	PPV	NPV	AUC	statistical method	References
2016	Karlsen	150	Danish	27.00%	262.00	–	–	–	–	0.79	logistic	1 [18]
2012	Angioli	57	Italy	66.70%	262.00	86.10%	89.50%	93.90%	77.00%	0.86	ROC	1 [19]
2012	Braicu	275	Germany	68.40%	235.00	76.60%	47.30%	–	–	0.63	ROC	1 [20]
2013	Braicu	275	Germany	68.50%	500.00	51.90%	70.40%	–	–	0.63	ROC	1 [20]
2016	Shen	39	China	–	353.22	77.40%	75.00%	92.30%	46.20%	0.76	ROC	1 [21]
2015	Tang	90	China	47.70%	473.00	81.00%	56.00%	67.00%	73.00%	0.72	ROC	1 [22]
2017	Paunovic	50	Serbia	44.00%	413.00	–	–	–	–	–	logistic	1 [23]
2014	Glaz	56	Poland	45.00%	218.43	86.60%;	91.30%	92.90%	84.00%	–	ROC	1 [24]

## Discussion

Recurrence of ovarian cancer after surgery and first line chemotherapy remains a problem. Recurrant oviarian cancer typically poorly responds to first, and sometimes even second line chemotherapy. In this scenario, it possible that the ovarian cancer inherent resistance may be due to reduced immunosurveillance and drug-resistant cells [18, 19]. Surgical removal of drug-resistant tumors can obtain the maximum clinical benefit. Recent advanced robotic approaches increase efficacy of safe surgical debulking of early-stage ovarian cancer. Currently the majority of patients with FIGO III-IV are staged by laparotomy [20, 21]. Non-invasive biomarkers and imaging indicators are the first choice for the prediction model of advanced ovarian cancer patients. Imaging combined with physical examination and health assessment is a common indicator for clinicians to judge whether advanced ovarian cancer patients can perform ideal tumor cytoreductive surgery. However, the systematic review shows that the CT-based predictive model does not have good sensitivity and specificity on surgical residual lesions and the CT model is complex and difficult to apply to clinical practice [22].

It has been thought in the past that higher preoperative serum CA125 levels are directly related to a larger tumor burden, and accurate CA125 cut-off values can help distinguish whether ovarian tumor patients can achieve optimal surgical cytoreduction. But based on studies over the past 10 years, there is no uniform conclusion as to whether preoperative CA125 level can predict primary optimal surgical cytoreduction [23–25]. Most studies have shown that 500 U/ml is the appropriate cut off value for CA125, but not practical in clinical applications. The study reported that the AUC of preoperative CA125 predictive optimal surgical cytoreduction was 0.57–0.83 [26–30], prediction efficiency is either weak or high [25, 31, 32]. There are few studies on HE4 in predicting primary optimal surgical cytoreduction of advanced ovarian cancer, but almost all studies assessing the predictive value of serum HE4 level in predicting surgical outcome for ovarian cancer have confirmed the effectiveness of HE4.

Our study shows that preoperative serum CA125 combined with preoperative serum HE4 can predict whether advanced ovarian cancer patients can achieve optimal surgical cytoreduction. The advantage of this study is that the latest standard RD = 0 is used as the definition of primary optimal surgical cytoreduction, only patients with advanced ovarian cancer are included, and predictive models have been developed from two different methods (PIV and logistic regression) for easy understanding and management. Important clinical variables were also considered: combined with preoperative serum CA125 and HE4. In fact, predictive models including age, FIGO stage, histological type, grade, preoperative serum CA125 level, and preoperative serum HE4 level were more accurate than the CA125 model alone.

Accumulating evidence suggests that the management of ovarian cancer should be personalized taking into account the performance status of the patient, in particular in case of elderly women [33, 34]. We included several important preoperative non-invasive parameters (age, FIGO stage, histological type, grade, preoperative serum CA125 level, preoperative serum HE4 level, ECOG performance status, and ASA). The accuracy of inclusion parameters in predicting whether advanced ovarian cancer patients can achieve optimal surgical cytoreduction is 0.49–0.68. The diagnostic accuracy, NPV, PPV, specificity of PIV model was 71, 100, 68, 100%. Negative predictive value represent a very important clinical parameter and are the proportion of patients who are considered to have unresectable disease and do not actually achieve optimal surgical cytoreduction. Negative predictive value of 100% can avoid unnecessary laparotomy exploration.

In 2006, Fagotti et al. [35] quantified the lesion information obtained under laparoscopic exploration, and predicted the statistical probability of the surgical outcome according to various factors, seven laparoscopic features were selected for inclusion in the PIV model. When PIV ≥ 8 points, the specificity, PPV, and NPV of the model was 100, 100, and 70%. This model has been validated later. However, laparoscopy also has certain limitations. In fact, as the disease advances, technical limits due to the absence of a direct tactile evaluation by palpation and the presence of fixed masses and carcinomatous adhesions hindering the visualization of certain anatomical spaces are intuitive. For patients who can not achieve optimal surgical cytoreduction, laparoscopic exploration exposes them to surgical complications, delays the onset of chemotherapy, and does not yield prognostic benefits.

Since Fagotti’s laparoscopic PIV model has a 100% positive predictive value, the negative predictive value of our study is 100%. The evaluation model of this study suggests that patients who can achieve optimal surgical cytoreduction may benefit from the second laparoscopic evaluation. Those patients who are judged to be resectable by the inexpensive, simple and non-invasive preoperative prediction model in our study can be re-evaluated by a more expensive and complex laparoscopic model. This secondary evaluation method can improve the expected surgical outcome and reduce the rate of inappropriate exploration. Similarly, the logistic model for predicting surgical outcome with age, preoperative serum CA125 level and preoperative serum HE4 level also has certain predictive efficacy. Like the PIV model, it confirms that the combination of preoperative serum CA125 level and preoperative serum HE4 level can establish a reliable preoperative non-invasive predictive model. This reliable non-invasive preoperative predictive model can be used before expensive and complex predictive methods such as laparoscopy or imaging.

There are several shortcomings in this study. First, like other models for predicting whether ovarian cancer patients can achieve optimal surgical cytoreduction, this study is a retrospective analysis. Second, the number of samples is small, and the model therefore needs further external validation. Third, resectability is strictly correlated to surgeons’ skills. This variable is difficult to ascertain and was not included in our model.

In conclusion, our study demonstrates in the initial treatment plan for advanced ovarian cancer patients, the diagnostic accuracy of age, preoperative serum CA125 level and preoperative serum HE4 level in assessing optimal surgical cytoreduction should be considered. They can be used as a pre-evaluation before laparoscopy or diagnostic imaging prediction models.

## Data Availability

Not applicable.
